# Adolescents' experiences of risk and protective factors in relation to mental wellbeing and mental health: a typology developed using ideal-type analysis

**DOI:** 10.3389/frcha.2025.1540343

**Published:** 2025-09-12

**Authors:** Mia Eisenstadt, Emily Stapley, Marisa Benedito, Amanda Junesing Chan, Athina-Marina Metaxa, Jessica Deighton

**Affiliations:** ^1^ Evidence Based Practice Unit (EBPU), Anna Freud National Centre for Children and Families and University College London (UCL), London, United Kingdom; ^2^ Department of Psychology, University of California, Berkeley, CA, United States; ^3^ Nuffield Department of Primary Care Health Sciences, University of Oxford, Oxford, United Kingdom

**Keywords:** wellbeing, protective factors, risk, stressors, adolescent, qualitative, typology, resilience

## Abstract

**Background:**

There is increased interest in adolescent wellbeing and the factors that increase or decrease the risk of mental health difficulties during adolescence. Extensive research exists for risk and protective factors, but few qualitative studies have been conducted in this area. Analysis of qualitative data can add insights into adolescents' perceptions and provide an opportunity to observe patterns in their subjective experiences.

**Objectives:**

The aim of this research was to explore patterns in adolescent-reported risk and protective factors in relation to the outcomes of mental wellbeing and mental health.

**Methods:**

The data for this study were drawn from interviews across five sites in England, conducted as part of the 5-year national evaluation of the HeadStart Programme. The sample comprised 63 adolescents aged 11–12 years from the first annual wave of qualitative data collection in 2017. Ideal-type analysis was used to construct a qualitative typology to delineate patterns in adolescents' experiences of risk and protective factors.

**Findings:**

Three distinct “types” or patterns of risk and protective factors in relation to adolescents' mental wellbeing and mental health were identified across the sample: the adolescent with “Uncertain Sources of Support,” the adolescent with “Self-Initiated Forms of Support,” and the adolescent with “Multiple Sources of Support.”

**Conclusions:**

Findings illustrate that distinct patterns exist in terms of adolescents' profiles of perceived risk and protective factors, with adolescents having clear differences in the levels of support that they perceived around them and the extent to which they felt that they could initiate, access, or find support to manage reported risk and stressors. These profiles may offer insight into the varied pathways through which adolescents attempt to navigate and manage threats to their mental wellbeing.

## Introduction

Adolescence is a significant stage of development marked by increasing independence, identity formation, and a shift from familial to peer and wider social influences (1, 2). During this period, adolescents encounter a range of new stressors and potential threats to their wellbeing, and begin to develop their own coping strategies (3–5). Although there is a substantial body of research on adolescent risk and protective factors in relation to mental health, wellbeing, and resilience (e.g., 6–9), less is known about how these factors are subjectively experienced and navigated by adolescents themselves. Gaining a deeper and more nuanced understanding of these lived experiences is important, as the way that adolescents perceive and respond to risk, in their own words, is a gap in extant research on adolescent resilience to adversity.

Further, given the important role of protective factors in reducing the likelihood of poor mental health and promoting adolescent mental wellbeing, exploring adolescents' experiences of protective factors can assist with identifying which and when adolescents may require additional support after exposure to risk and stressors (10–14). Fostering resilience during the adolescent period is important, as adolescents encounter new stressors during a period of rapid development, which can pose increased risk for the onset of psychopathology and poor health outcomes (15, 16). During this time of increasing autonomy, adolescents form closer bonds with peers outside of the family; however, research suggests that adolescents' sense of connectedness and supportive relationships with parents and carers remain important for positive mental health and wellbeing (17).

Within the study of resilience, theorists have noted a striking absence of research featuring children and adolescents' views on their own wellbeing and contributors therein (18, 19). Thus, there is a need to better understand the ways that children and adolescents describe the factors that contribute to their mental health and wellbeing. Further, by qualitatively studying the potential patterns in the ways in which adolescents describe their overall profiles of risk and protective factors, it is possible to identify adolescents who may experience co-occurring factors and may have least protection. An additional gap in existing literature is the reliance on variable-centred approaches that examine relationships between multiple risk and protective factors in predicting mental health and wellbeing outcomes. Although this approach can yield useful associations, adopting a person-centred approach may offer a richer and more nuanced understanding of adolescents' experiences. Qualitative studies can potentially illuminate individual experiences, without limiting responses to pre-conceived categories (20); it can help researchers to understand what risk factors and protective factors feel like and how they are subjectively experienced in adolescents' day-to-day lives in their own words (21). Further, qualitative studies take an idiographic approach that is concerned with the unique characteristics of each individual adolescent versus generalised characteristics or variables (22). Person-centred studies on risk and protective factors are an emerging area of research on adolescent mental health (for recent studies see (23, 24)).

Nevertheless, person-centred methods have been applied to yield helpful patterns and types. Studies group individuals based on shared profiles using quantitative analysis techniques, such as cluster analysis and growth mixture modelling, to categorise youth according to their exposure to risks and the presence of protective assets (25–27). These approaches have proven valuable in constructing typologies of risk and protective factors and resilience, particularly in research contexts dominated by variable-focused models (25, 28, 29). For instance, Solberg et al. (26) examined whether combinations of self-efficacy, internal motivation, family support, peer and teacher connections, and exposure to violence were associated with different academic outcomes in a sample of 758 predominantly Latino and African American adolescents aged 13–17 years. Their analysis yielded six distinct clusters, ranging from “most vulnerable” to “not at risk.” The most vulnerable group reported significantly lower levels of motivation, peer and teacher support, family support, and self-efficacy, alongside greater exposure to violence.

Another example typology of adolescent risk and protective factors was constructed through conducting a latent profile analysis, a type of structural equation modelling, which linked community violence, protective factors (self-worth, parental monitoring, and parental involvement), and adolescent mental health as an outcome (25). The study found three categories: “vulnerable,” referring to a class of adolescents with the highest levels of exposure to violence and low levels of protection; the second type, “moderate risk/medium protection,” reported lower risk but had increased self-worth; and third, the “moderate risk/high protection” group also reported lower risk but had the highest level of positive self-perceptions in the sample (25). Notably, there was no category for a low-risk/high-protection group in this context.

Ungar et al. (27) conducted a mixed methods study of 85 adolescents receiving support from therapeutic relationships in Canada. The study involved qualitative research interviews and the calculation of a risk and resilience score for each participant through their completion of a range of measures. Subsequently, four types were identified from the quantitative aspect of the research, comprising “high-risk, high-resilience,” “high-risk, low-resilience,” “low-risk, low-resilience,” and “low-risk, high-resilience” adolescents (27). The qualitative interviews revealed that adolescents in the “high-risk, low-resilience” group preferred informal support. In contrast, adolescents with higher resilience scores described helpful therapeutic relationships that were structured with clear boundaries (27). This typology provides an example of how patterns in risk and resilience can provide insight into discernible differences in adolescents' preferences regarding support. Although this study took a mixed methods approach to exploring adolescents' risk and resilience profiles, to date there has been a lack of use of qualitative methods to explore this.

The current paper aims to add to this emerging research and to address the lack of detailed insight thus far around the perceptions and experiences of adolescents of risk and protective factors. Specifically, the aim of this study was to take a typology development approach to examine patterns qualitatively in adolescents' experiences of risk and protective factors in England. The main focus was the range of reported risk factors that adolescents experience and the protective factors or support that they perceive to be in place in relation to risks.

For the purpose of the current study, the following definitions have been adopted. A risk factor has been defined as “a characteristic, experience, or event that, if present, is associated with an increase in the probability (risk) of a particular outcome over the base rate of the outcome in the general (unexposed) population” (30). A protective factor has been defined as a variable that may change, interact with, improve, or influence an outcome, either in the context of a known risk factor or not. This draws on the definition by Kazdin and colleagues that protective factors refer to “antecedent conditions associated with a decrease in the likelihood of undesirable outcomes or with an increase in the likelihood of positive outcomes” (30). In this study, protective factors have been understood to be variables that decrease the likelihood of the outcomes of poor mental wellbeing and/or psychopathology and increase the likelihood of positive mental wellbeing and the absence of psychopathology.

## Methods

### Setting for the study

The data for this study were drawn from interviews conducted as part of the qualitative research strand of the national evaluation of the HeadStart Programme in England (e.g., 4, 5, 19, 23). HeadStart was a 6-year initiative funded by The National Lottery Community Fund. The programme aimed to identify and test innovative approaches to enhancing the mental health and wellbeing of adolescents aged 10–16 years, while also seeking to prevent the emergence of severe mental health challenges. This objective was pursued through six HeadStart partnerships led by local authorities in Blackpool, Cornwall, Hull, Kent, Newham, and Wolverhampton (the research began in five sites and was later implemented in a sixth site). These partnerships collaborated extensively with children and adolescents, schools, families, charities, community organisations, and public services to integrate mental health and wellbeing as a shared community responsibility (31).

### Participants

Interviews were conducted with 63 adolescents at the first timepoint of data collection for the qualitative research strand of the national evaluation of the HeadStart Programme (May to July 2017). A total of 63 participants were drawn from 14 schools across five HeadStart areas. One school participated from HeadStart Area 1, contributing 12 participants. Two schools in Area 2 contributed a total of 14 participants (eight and six, respectively). In Area 3, two schools participated, providing four and two participants. Area 4 included two schools, contributing seven and eight participants. Area 5 involved three schools, contributing five, three, and eight participants, respectively. At this point in the evaluation of HeadStart, only five of the six partnerships were ready to have interviews conducted in their area (one area was still in the programme preparation phase). School or HeadStart staff invited adolescents to take part in the interviews if they were eligible to receive universal (whole class) or targeted (individual or small-group) support through the HeadStart programme. Participants self-reported their demographic information. The sample included 28 girls (44.44%) and 35 boys (55.55%). Participants' ages were in the range of 9.10–12.9 years (*M* = 11.90, *SD* = 0.59). Ethnicity data for the sample are shown in Table 1.

**Table 1:** Total number and percentage of participants from different ethnic groups.

Ethnicity	Total in time 1 (%)
Asian or Asian British	5 (7.94%)
Black or Black British	3 (4.76%)
Mixed Ethnic background	6 (9.52%)
White or White British	48 (76.19%)
Any other ethnic background	1 (1.59%)

Demographic data for time 1 (2017) of qualitative longitudinal study (2018).

### Ethical considerations

University College London granted ethical approval for the qualitative research strand of the national evaluation of the HeadStart Programme (ID number: 7963/002). Adolescents were given the option of participating in the interviews, which they could accept or decline. Before being interviewed, participants and their parents or carers were invited to read a participant information sheet outlining the study. Then, informed consent was obtained from parents and carers, and participants' assent was obtained before the interviews began. Interviewees were informed that the information that they provided would be kept private unless they revealed something that indicated risk to themselves or others. During the transcription phase, all identifiable information was anonymised.

### Data collection

All interviews were conducted in private rooms at the participants' schools. Semi-structured interviews were chosen to allow participants to provide free-flowing answers (32), with the interviewers guided by an interview schedule. The interview schedule was developed collaboratively with the research team and Common Room, a youth advocacy organisation. The length of the semi-structured interviews conducted with participants was in the range of approximately 15–60 min (*M* = 39.73 min, *SD* = 10.33). Interviews were transcribed verbatim. Identifying details, such as personal names and the names of specific places, were altered to protect participant anonymity. The interviews, conducted as part of the HeadStart Programme national evaluation, were designed for young people and did not use the terms “risk” or “protective factors.” Instead, they explored adolescents' experiences of life challenges, coping strategies, sources of support, and what contributed to their happiness, thus providing qualitative insights into adolescents' perceptions and experiences of protective factors and associated risks.

### Data analysis

Ideal-type analysis was used to qualitatively explore adolescents' experiences of risk and protective factors, and to understand patterns of adolescent-reported risk and protective factors in relation to mental wellbeing and mental health. Ideal-type analysis is derived from a concept developed by the sociologist Max Weber (33) and was later developed as a qualitative methodology by Gerhardt (34). In a qualitative research context, an ideal type is a *theoretical construct* that represents a simplified, internally consistent version of a pattern found across multiple participants' experiences (35). Ideal-type analysis is a systematic process of comparison and contrasting all of the cases or participants in a dataset until general mutually exclusive categories or patterns of common characteristics become apparent to the researcher (35, 36). The method for ideal-type analysis used in this study drew on the seven steps outlined by Stapley et al. (35, 37).

#### Step 1: Familiarisation with the dataset

The first author read all of the transcripts and took notes and made observations about adolescents' descriptions of risk and protective factors, based on the set of definitions assigned for the purpose of this study (see introduction for reference). The second author also read through the dataset to check that the first author's notes and observations reflected the data.

#### Step 2: Development of individual case reconstructions

A case reconstruction (description of the transcript content) was drafted from each transcript by the first author with a narrative of the reported risk and protective factors from each transcript. Each case reconstruction was printed onto paper.

#### Step 3: Constructing the ideal types

To detect patterns in the data, each case reconstruction was rigorously compared and contrasted with the other case reconstructions. Participants whose qualitative accounts of their experiences shared similarities were grouped together to form “ideal types” or groupings of participants with shared experiences (35). Due to the large dataset, the comparing and contrasting was initially undergone with half of the sample of cases (*n* = 31). This was performed by the first author, with developing type names and descriptions shared with the second author. The second author then independently grouped the cases according to the type name and description. After discussion and review of this process, the first author edited the descriptions of the ideal types to prepare for the next step.

#### Step 4: Selecting optimal cases

Optimal cases were selected through reading the case constructions within each ideal type. The optimal case is the example that best captures the core pattern shared by similar cases within a group. It acts as a reference point, allowing the researcher to compare other cases in the same type and assess how closely they align with it (35). All remaining cases in the dataset (*n* = 32) were then clustered around the relevant optimal case, that is the case that was most similar to them. This was undertaken by a systematic process of “sorting and forming,” where cases were matched with other cases that were most similar (36). An audit trail documenting the rationale for including each case within each ideal type was maintained in an Excel spreadsheet throughout the analysis process.

Each case was categorised into one of the ideal types based on the type descriptions, with the exception of two cases. The data in these cases were much less rich and detailed, rendering them difficult to classify into one of the types, but the cases themselves were not sufficiently distinct to warrant additional type names. Discussions were held with the second and last authors until a consensus was reached on the categorisation of these two cases into the existing types.

#### Step 5: Forming the ideal type descriptions

With the optimal case for each type in mind, a comprehensive description of each ideal type was developed. The cases categorised within each type were represented to different extents by the descriptions but shared the key features that exemplified that type (38). Each type was given a heading and a description drawing on participants' own use of language where possible.

#### Step 6: Credibility and consistency checks

The last author then independently grouped all cases according to the type name and description, as an additional form of credibility check on the analysis (36). The last author had limited involvement in the steps of the ideal-type analysis methodology until this point (they had not read the ideal type descriptions nor the case reconstructions) and was thus considered independent. This step is not intended to measure interrater reliability (as per (35)), but rather to check that the ideal type descriptions are well grounded in the data and sufficiently clear and distinct.

All of the researchers involved in conducting interviews and writing the case reconstructions actively discussed the biases that they brought to the research process from their subjective positions and sources of privilege. The first author kept field notes of biases and assumptions throughout the analysis and reflections on the interview technique after each interview. Differences in interpretations of risk and protective factors were discussed by the first, second, and last authors.

Input and further detailed review were also sought from an external researcher who reviewed the type names and descriptions and considered whether each type contained cases that shared similar characteristics, and further still, if the three types were sufficiently distinct. Furthermore, as the types aimed to represent adolescents' qualitative data, a young advisor provided feedback on the type names and the language of the ideal type descriptions from a young person's perspective. In the initial type descriptions, the young advisor suggested that the authors ought to be careful that the types did not sound deterministic towards a negative outcome, especially for young people with high levels of risk factors. For example, one of the types was initially given the name of “The adolescent on shaky ground,” which the young advisor felt implied a negative outcome. However, within the data, this was not always the case. Therefore, in response to this, this type name was changed to “The adolescent with ‘Uncertain sources of support.’” The type names and descriptions were subsequently reviewed by the fourth and fifth authors for further refinement.

#### Step 7: Making comparisons

Stronger and weaker examples within each ideal type, in terms of the degree to which participants' qualitative accounts reflected the ideal type descriptions, were identified to understand the homogeneity and heterogeneity within each type as part of the write-up of the typology. Stronger examples had a closer resemblance to the optimal case. Weaker examples had less similar characteristics to the respective optimal case, but nonetheless still represented the ideal type description.

## Results

**Figure 1:**
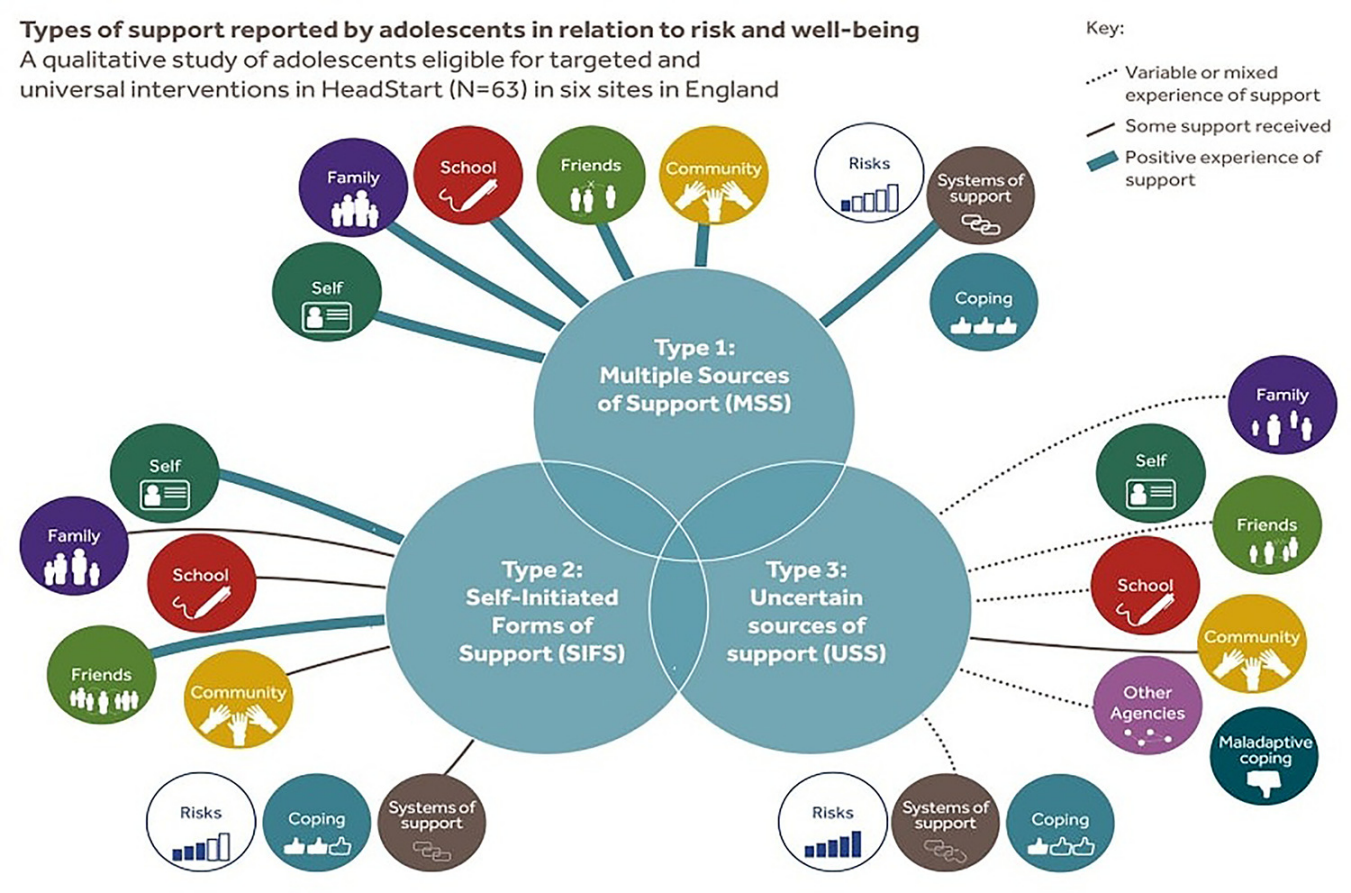
A typology of categories of support in relation to risk and adolescent wellbeing.

Three ideal types of reported risk and protective factors in relation to mental wellbeing and mental health were developed through the application of ideal-type analysis. The types were: the adolescent with “Uncertain Sources of Support” (USS), the adolescent with “Self-Initiated Forms of Support” (SIFS), and the adolescent with “Multiple Sources of Support” (MSS). The number of participants (including a breakdown by sex) assigned to each type is provided in Table 2. The most common type was the USS adolescent (*N* = 35) and the least common was the SIFS adolescent (*N* *=* 7). A description of each ideal type is provided, together with a description of the optimal case for each type. An overview of the types of support reported by adolescents in each ideal type can be seen in Figure 1.

**Table 2:** Number and percentage of participants in the three ideal types, by number of boys and girls.

Gender	The adolescent with “Uncertain Sources of Support’” *N* (%)	The adolescent with “Self-Initiated Forms of Support” *N* (%)	The adolescent with “Multiple Sources of Support’” *N* (%)
Boys	18 (51.43%)	5 (71.43%)	12 (57.14%)
Girls	17 (48.57%)	2 (28.57%)	9 (42.86%)
Total	35 (55.56%)	7 (11.11%)	21 (33.33%)

The first two rows show the number *N* (%) of boys and girls within each type; the bottom row shows the number (%) of adolescents belonging to each ideal type out of the total sample (*N* = 63).

### Ideal type 1: The adolescent with “Uncertain Sources of Support”

The USS type referred to adolescents who reported few protective factors, which were not always perceived to alleviate the effects of a risk or stressor or which were described as inconsistent, ambiguous, or absent. A total of 35 cases (55.55% of the total sample) represented this type. The number of boys found in this type was slightly higher than the number of girls: *N* = 18 (51.42%) compared to *N* = 17 (48.57%).

#### Ideal type description

Adolescents in the USS type tended to describe having few protective factors or sources of support in the context of multiple reported risks or sources of stress. Some factors were reported as protective at times, and at other times were described as a cause of difficulty in themselves, such as a difficult, though sometimes supportive, relationship with a parent. Some coping strategies were described by adolescents in this type that would potentially constitute styles of maladaptive coping, such as avoiding the stressor or using a distraction.

Some severe and/or chronic types of risk were described, with the precise range of risks and stressors unique to each participant. Some of the adolescents in the USS type experienced a high number of risks and stressors (more than three or four, as can be seen in the optimal case) and a lack of protective factors. Adolescents in the USS type often reported a range of risk factors that are established correlates of poor wellbeing or risk of mental health disorder. Individual risk factors included behaviour issues, difficulties managing thoughts and emotions, and worry. Family risk factors included parental unemployment, family poverty, parental ill-health, conflict with parents, difficulties in relation to parental separation and family transitions, and conflict with siblings. Community and school risk factors could include conflict with friends and peers (e.g., negative peer influence, bullying), difficulties with teachers, school exclusion or risk of exclusion, difficulties with schoolwork, and worries about school.

### Optimal case: “Freddy”

Freddy described how his parents' separation, which occurred when he was much younger, was a present-day source of unhappiness in his interview. Freddy reported receiving physical discipline from both of his parents. However, Freddy also described having a “happy relationship” with his father. He spoke about his relationship with his mother more negatively and expressed that there was a lack of “respect” in his relationship with his mother, who criticises him for “talking smart.” He shared that he felt threatened by his mother's current partner, whom he believes is allied with his mother against him in arguments. Freddy also finds his relationship with his sisters to be strained, stating that he does not get much support with “being a boy” at his mother's house.

To receive support in relation to risks at home, Freddy has a range of people that he could call—notably his grandmothers, his father, and his friends—but he is reluctant to ask for help due to his fear of burdening others. He described, “To be honest, I don’t wanna be putting my own problems on other people. …it's something that I have to deal with myself, and if… I really do need help… I’ll know who to get it from.” Freddy reported a few activities that contribute to his happiness: his enjoyment of colours through art, watching TV, and “chilling” with his cat. Freddy expressed difficulty with managing his feelings, either holding a toy when he is sad or “crying out” his feelings.

When Freddy cries at school, he is sometimes mocked by his peers, which can lead to fights and conflict. Freddy is part of a group of friends who are “smart,” but also “troublemakers.” He values this group but worries about being excluded from school for his bad behaviour. He appreciates having the opportunity to share his private thoughts and feelings with a peer mentor (an older student) who he has recently met through HeadStart, especially after having been teased at school.

### Other cases in the USS type

Cases strongly reflecting this type were those participants who, like Freddy, stressed ambiguity in the support that they received or could access, or who reported a perceived absence of support in relation to risks. For example, Michael reported a recent experience at school in which he was being bullied and stated that he was reluctant to seek help from his parents as his parents knew the bully's parents. This case differed from the optimal case because there was an ambivalence in terms of the parental support available to him in terms of alleviating the stressor (bullying). This contrasts with the optimal case who reports his mother as a source of stress and also reports receiving physical discipline from both of his parents.

In other cases (i.e., those who were less similar to the optimal case but who nonetheless still represented this type), an adolescent may have had a source of support or protection that alleviated some effects of a risk or stressor, but it was ambiguous as to whether the support was effective and if the adolescent's overall profile of protective factors was sufficient in relation to managing the risks and stressors that they faced. In addition, sometimes there were elements of protective factors that were perceived as helpful by these cases. For example, in Thomas' case, he reported a difficult custody battle between his separated parents and struggling with learning difficulties, but close relationships with his parents and immediate family. In his interview, he indicated on the one hand that support for his learning difficulties was ineffective: “She made all the things difficult.” However, he also expressed on the other hand that his support worker's strict discipline had helped him with sitting still: “I was always getting told off by the teachers saying, ‘Sit still. Other people can do it, why can't you?’ So, she sort of, kind of, did help me sit still.” In this case, Thomas appeared unsure if the support with his learning difficulties had helped, he struggled with his parents' separation, but he also felt connected to his parents, and so the extent that his protective factors overall are effective is ambiguous. This ambiguity or uncertainty is a characteristic of this type; however, this case is different from the optimal case in that he has a close connection with his parents, whereas the optimal case reports parental maltreatment and a difficult relationship with his mother.

### Ideal type 2: The adolescent with “Self-Initiated Forms of Support”

This type referred to adolescents who predominantly described a range of “self-initiated” protective factors in relation to their mental wellbeing and mental health, such as coping strategies and their own qualities. A total of seven cases represented this type (11.11% of the overall sample), including 5 (71.42%) boys and 2 (28.57%) girls.

#### Ideal type description

Adolescents in the SIFS type tended to emphasise their own role in coping with the negative effects of risks instead of drawing on support from parents or school. Adolescents may in fact support others, rather than receive support themselves. Self-initiated strategies included engagement in leisure activities (e.g., participation in sport, listening to music, or playing games) and providing emotional or financial support to family members, friends, or themselves.

Adolescents in the SIFS ideal type sometimes reported receiving some limited support from sources such as parents, friends, family, school, or wellbeing interventions at school (e.g., HeadStart). Cases in this category more often tended to reflect on their own coping capacities at the individual level (e.g., traits such as being mature or independent) or drawing on inner resources (e.g., self-control, maturity, or concentration skills). Some adolescents classified as SIFS referenced ambitions they are achieving in addition to school, including advancing in extracurricular activities like sports, music, or a career goal.

SIFS adolescents experienced similar types of risk factors reported in the previous type, this included family poverty, arguments with siblings, peer conflicts, parental ill-health, worries about parents, difficulties with schoolwork, and relationship issues with parents. However, discussions of risk were often less severe than in the USS type and, in general, they do not mention as many risks as the previous type. Adolescents designated as SIFS described having sufficient support in place to manage risks—for the most part, they manage risk themselves. Belonging to this type did not entail being protected against all risks, but it did entail that a participant reported perceiving themselves to be able to cope with risks and having a range of resources that are primarily internal (e.g., maturity) and secondarily external (e.g., parents) to draw on.

### Optimal case: “Jamal”

Jamal described himself as a “bright kid” who generally does well at school. Outside of school, he is a semi-professional sports player and consequently has an income stream that he can contribute towards the household, which is helpful given his parents' circumstances. However, he reported that having a job outside of school raises some additional challenges, such as missing classes and having less time for homework. At school, Jamal reported that he feels that the experience can be “disheartening” if he does not achieve the grades that he hoped to achieve.

Jamal reported that he dealt with problems primarily by himself, only seeking support from his mother if things became “overwhelming.” He described his relationship with his mother as close, in that he feels able to tell her anything. Jamal explained that his mother then relays information to his father because she understands everything. Jamal felt that sometimes his mother shared things with him that he felt were not always appropriate to share with a child, but that he felt that this had contributed to him maturing earlier.

#### Other cases in the SIFS type

Cases strongly reflecting this type were participants who referred to a pre-eminence of self-initiated strategies in relation to risks (rather than drawing on support from others). For instance, Doug described his primary protective factor as spending time outside with his friends to help him relax and manage problems at home.

Other cases (i.e., those that were less similar to the optimal case but who nonetheless still represented this type) in the SIFS ideal type either described only a few or less effective self-initiated coping strategies. They were designated as SIFS because they employed self-generated support rather than turning towards adults or peers, but unlike the optimal case, their strategies had mixed results in alleviating difficult emotions or reducing a risk or stressor.

### Ideal type 3: The adolescent with “Multiple Sources of Support”

Adolescents in this third type reported receiving a range of effective support from school, parents, and/or other external sources in either the presence or absence of some reported risks. A total of 21 cases (35% of total sample) represented this type, which comprised 12 (57.14%) male adolescents and 9 (42.85%) female adolescents.

#### Ideal type description

Adolescents in the MSS type were characterised by reports of using a variety of types of support or a prominent type of support from their parents, community, and peers. Some examples of support also included HeadStart interventions, mental health services or social services, extracurricular activities (e.g., sports, creative activities), and places of worship. Participants in this type reported support sources as positively associated with increased mental wellbeing, such as feeling happiest spending time with family. Other protective factors included those that were perceived to be effective at removing or reducing a stressor, such as a teacher resolving bullying at school or receiving assistance with difficult schoolwork.

In contrast to the USS type (where adolescents described more severe and chronic risks, such as prolonged bullying, ongoing conflict with a caregiver and maltreatment, or difficulties managing anger) participants in the MSS type typically reported fewer and less severe risks. This group appeared to experience a relatively stable and manageable day-to-day context, at least from their own perspective. Thus, the MSS type was characterised by adolescents who reported experiencing either minor stressors, in their view, or no risk factors at all. Examples of minor stressors included occasional sibling disagreements, challenges in a specific school subject, or disliking a particular teacher. In some cases, participants explicitly stated an absence of stress, using phrases such as “I have no problems in my life” or “I am happy with my life,” which were taken to indicate a sense of overall wellbeing.

### Optimal case: “Isobel”

Isobel reported that she generally enjoyed school and succeeded academically but, at times, felt bogged down by exam pressure. She feels like she has achieved something if she has helped another person with a subject in which she is proficient. She reported careful attention towards her schoolwork, which she credits to a teacher early on in primary school. In addition to school, Isobel described how participating in an ongoing school extracurricular activity gave her a sense of pride and belonging.

In terms of life at home, Isobel reported a positive experience of family life. She viewed her parents as supportive of her as a person, her schoolwork, and her extracurricular activities. Isobel described that she talks to her mother about her daily life, and if her mother is busy, she would talk to her father or her friends, highlighting the importance of social support.

Regarding handling difficult situations, Isobel described that sometimes at home she gets angry and then will go up to her room and be quiet on her own. She sometimes receives disciplinary consequences from school for not doing her homework. When she comes across a peer conflict, Isobel reported that she finds it difficult, but she handles it by avoiding or trying to resolve it. Isobel reported that she found a HeadStart peer mentor (an older student with whom she met periodically to provide support and problem-solving as part of the in-school HeadStart support) helpful for sharing instances when she got into trouble at school and did not want to share it with her mother. Overall, Isobel reported that she did not have any “big problems” in her life and that receiving mentoring had helped her to have more confidence.

#### Other cases in the MSS type

Cases strongly reflecting this type reported effective support from several of the following sources: parents, siblings, school staff (including pastoral staff and/or teachers or headteachers), friendships, staff linked with extracurricular activities, adults belonging to a religious institution, or staff from the wider community and individuals related to HeadStart interventions. Participants in the MSS type referred to their own strengths, such as managing to regulate their own emotions, and having generally effective coping strategies and support in relation to difficulties.

Other cases (i.e., those that were less similar to the optimal case but who nonetheless still represented this type) were those for whom there were either not many sources of support or wherein some of the sources of support were mixed in terms of their efficacy; some were effective in reducing the harmful effects of stressors, but others were not. For example, Sam discussed finding some external support through HeadStart interventions he had received as helpful and enjoying spending time with family. Yet, Sam also described struggling with disruptive peers at school and had recently ended a friendship with a boy who was increasingly involved in deviant activities. Sam felt that he could not speak with his parents about the behaviours of his peers at school because doing so might cause undue “worry” to his mother. Sam was designated as having multiple forms of support due to a relatively high level of parental support and other external support, but because the support of his parents was perceived as limited, he is less similar to the optimal case.

## Discussion

This study has presented a typology consisting of three types: The adolescent with “Uncertain Sources of Support,” the adolescent with “Self-Initiated Forms of Support,” and the adolescent with “Multiple Sources of Support.” The typology was developed using qualitative data from 63 adolescents in England.

The USS type was the most prevalent ideal type in the sample. The USS type refers to adolescents who reported protective factors in relation to risks as being absent, variable, or serving as additional sources of risk. Members of this group reported a high number of risks and limited support and coping strategies. Although some protective factors may be in place, it is uncertain if they are effective at countering risk. When comparing the USS type to other typologies of protective factors, due to the number of stressors that can be construed as risk factors reported by the USS adolescents (e.g., behaviour issues, difficulties regulating emotions, interparental conflict, low socioeconomic status), this type resembles the “high-risk” category frequently used in extant typologies, where risk is ranked from low to high (39). The USS category might also resemble the “vulnerable” category in other typologies, which refers to young people with an absence of support from parents, school, and peers (25, 26).

However, in the USS type, a distinction is also made from “high-risk” categories found in other typologies because the emphasis is not only on the amount of risk (which is often high) but also on the perceived quality of support in relation to risks. Within the USS ideal type, support is reported as either absent or ineffective to counter the negative effects of multiple reported risk factors.

Resilience research has found that both “informal” and “formal” support systems foster resilience and reduce risk of psychopathology (40, 41). Informal support systems refer to family, extended family, and friends. Formal support refers to institutions, such as mental health interventions and social services who provide support when informal support systems are lacking (40). For USS adolescents, there is a lack of available support in both informal and formal systems around adolescents, as well as in the adolescent's capacity to negotiate resources from such systems.

Moreover, some factors—such as parents, other caregivers, service providers, and friends—can be sources of both support and sources of risk or possible harm for adolescents in the USS group (e.g., through a lack of support and connection with parents). Other studies have found that sources perceived as protective factors can also under some circumstances be a source of risk. For instance, a qualitative study with Australian female adolescents found that peers could be dually sources of emotional support and influence adolescents to engage in deviant behaviour (42). This is a parallel with the USS group where, for example, parental support could be at times protective and at times a source of risk with instances of child maltreatment.

The second most prevalent type found in the dataset, the MSS type, comprised 12 boys and nine girls. The MSS type was generally in agreement with extant literature that links increased support to greater mental wellbeing (40, 43). Support has been conceptualised as those acts that meet an individual's needs (44, 45). Participants within the MSS type discussed having positive, supportive relationships with their parents and other family members. This reflects previous findings that parents, in particular, can be key helpers and can provide support that is dependable and durable, which is important for child and adolescent wellbeing (17, 43, 45). For MSS adolescents, family support was also discussed in addition to a range of social support around them, which is understood to decrease risk of mental health disorder in adolescents and positively linked to wellbeing (41).

When compared with other typologies, the MSS type bears some resemblance to the “moderate risk/high protection type” found in other typologies of risk and protection. For example, in the study by Copeland-Linder et al. (25), the “moderate-risk/high-protection type” adolescents were found to be exposed to a degree of risk but have high levels of external and internal protective factors to counteract risk (such as high levels of parental support). The MSS type differs, however, in that there is a low level of risk for this subset of adolescents. The MSS type potentially reflects models in the literature that posit that greater numbers of protective factors better protect adolescents against risk and that there are added benefits that result from cumulative protection (12, 13, 46).

The least prevalent type identified in this study was the SIFS type, comprising five boys and only two girls. SIFS adolescents tended to draw on their own resources in coping, problem-solving, and managing stressors. The SIFS type concurred with current literature on resilience in some ways and diverged from it in others. For instance, on the one hand, SIFS can be likened to the “resilient” type found in other typologies (26, 27). For example, in the typology by Solberg et al. (26), the “resilient” category described adolescents who had higher levels of family support and self-efficacy in the context of exposure to violence. SIFS is comparable to the “resilient” type because the SIFS type had high levels of self-efficacy and more exposure to risk than the MSS type in the current study. However, adolescents in the SIFS type were not characterised by reported higher family support and so in this respect are quite different; instead, levels of family support varied. Nonetheless, their coping behaviours were seemingly effective from their perspectives, in contrast to adolescents in the USS type.

The autonomous aspect of help-seeking and problem-solving of the SIFS type has some similarities with the “low-risk, low-resilience” type that tended to avoid support that was provided to them identified in the study by Ungar et al. (27). Some adolescents in the SIFS type in our study did not take up school-based support or help from adults and did not report perceiving support provided to them as beneficial. Existing research has also reported that sometimes mental health support provision, such as therapy, may be unwanted, unhelpful, or resisted by adolescents (27, 47), and that adolescents may not seek help to cope with stressors (48, 49).

Within the SIFS type, sex differences were most marked: the majority were boys. This finding potentially corresponds with studies that suggest that girls are more likely to engage in help-seeking behaviour than boys (48, 50). More boys within the SIFS type could also reflect previous research that has found that boys are more likely to engage in individualised action towards the stressor (problem-solving alone) or avoidance coping (avoiding the stressor) (51), with girls more likely to access social support (1).

### Implications for researchers

Some studies of resilience examine the presence of protective factors through questionnaires, rather than perceived or qualitatively reported protective factors by adolescents themselves (52, 53). It is important to explore what support adolescents perceive as risk-reducing or as helping them to manage stressors. The complex profiles and variations in support-seeking (external) and autonomous problem-solving (internal) found here suggest the importance of understanding the diverse ways adolescents seek support from different sources (self, family, peers, school, community), as well as the extent to which each individual perceives effective support and protection as available. Further, it is relevant to examine adolescent perspectives on their internal, as well as external, resources.

Furthermore, the three ideal types or overarching patterns of adolescents' experiences of risk and protective factors identified here provide a possible new emphasis when considering the use of the language of risk. In many current resilience studies, the focus is on the adolescent's level of risk (high, medium, low) (54–57). The shift that our study suggests is from a focus on the level of risk to a focus on adolescents' subjectively perceived experiences of support and coping. For example, this would involve asking adolescents what they think about the support that they receive (if available) and how it helps them manage stressors and challenges, thereby capturing their subjective evaluations of that support.

The study also lends support to the argument (58) that designating adolescents as “high risk” may not be appropriate language to use, as it is too far removed from the language that adolescents use themselves. Researchers have also noted that the language of “high risk” can be deterministic regarding negative outcomes in the areas of mental wellbeing and mental health (58).

### Implications for support providers

Clear individual differences were found in three profiles of risk and protective factors experienced by each adolescent in our study. The specific needs of the three groups highlights the importance for intervention designers to consider that adolescents may cope with risks and stressors differently and may be highly attuned to whether support offered is helpful or appropriate for them individually (vs. peer support or their own strategies). Moreover, some adolescents may prefer to rely on their own coping strategies (than from external systems), while others are more inclined to seek support from parents or institutions, or may simply have access to support systems that are better suited to their needs.

The typology has the potential to serve as a framework to guide a dialogue with adolescents about what support they perceive to be available to them, and their perceptions and understandings of what types of protective factors reduce their difficulties in life (4, 5). This could then be used by professionals around an adolescent to understand their unique context that may contribute to presenting symptoms or behaviour at school, and to consider carefully what types of support may be more or less likely to be helpful for a young person. For example, an adolescent who reflects the MSS type may benefit from additional support in the form of short-term, universal interventions that build on existing support; an adolescent with SIFS may prefer support that they can access individually or choices in terms of when and how they receive support; and an adolescent with USS may require long-term interventions that support their family's underlying needs and help reduce stressors within their broader environment.

### Limitations

Participants' recall of past events may be influenced by factors such as mood at the time of reporting and social desirability bias, or the tendency of participants to provide anticipated socially desirable responses to questions, rather than those that more accurately represent their actual experiences (59, 60). The interviews are also a single snapshot in time and so participants' reports may vary day to day. Additionally, this study consisted of a secondary data analysis of interviews. It did not aim to collect reports from other sources about all the types of risk and protective factors in an adolescent's life. Further, future studies, triangulation with parent and teacher reports, and other data sources on risk factors could strengthen the validity of the results. It is thus understood that the findings presented here do not necessarily represent adolescents' entire experience of risk and protective factors. A related point is that participants could have been experiencing risks that were too sensitive to discuss with the researchers. Such difficulties might be absent from the data, analysis, and type allocation. The implication of this is that other sources of data about the adolescent would be required in the precise assessment of risk assessment, needs, and support provision. Another limitation is that without further confirmatory evidence of these types applied to other groups of adolescents, it would be difficult to generalise the findings from this study to other samples. Qualitative research is not expected to be generalisable in the statistical meaning of using a representative sample to apply results to a broader population (61, 62). However, scope remains for other types of transferability; for instance, using the typology with the same sample longitudinally or exploring the typology in other research contexts (62). For example, there are opportunities to conduct follow-up studies using the typology in subsequent time points of the HeadStart longitudinal study and furthermore inclusion of the sixth site after data collection in the sixth HeadStart area.

## Conclusion

This study applied ideal-type analysis, a person-centred, qualitative approach, to form three ideal types or overarching patterns of adolescent-reported risk and protective factors in relation to mental wellbeing and mental health. The typology highlights the variation in adolescents' different perceptions and experiences of their reported risk and protective factors. The typology shifts the emphasis from not only the number of risk and protective factors, or the level of risk and resilience perceived and experienced by adolescents, towards the perceived quality and experience of protective factors in relation to risk in their everyday lives, taking an adolescent-centred qualitative approach. In this way, our study aims to close the gap in our knowledge between adolescents' subjective experiences of support and protection, as sources of support that may be assumed to be protective may not be experienced as such by an adolescent.

## Data Availability

The raw data supporting the conclusions of this article will be made available by the authors, without undue reservation.

## References

[1] Branje S De Moor EL Spitzer J Becht AI. Dynamics of identity development in adolescence: a decade in review. J Res Adolesc. (2021) 31(4):908–27. 10.1111/jora.12678 34820948 PMC9298910

[2] Steinberg LD. Age of Opportunity: Lessons from the New Science of Adolescence. Boston, MA and New York, NY: Houghton Mifflin Harcourt (2014).

[3] Eisenstadt M Stapley E Deighton J Wolpert M. Adolescent stressors and their perceived effects on mental well-being: a qualitative study. Clin Child Psychol Psychiatry. (2020) 25(2):470–84. 10.1177/1359104519893314

[4] Stapley E Demkowicz O Eisenstadt M Wolpert M Deighton J. Coping with the stresses of daily life in England: a qualitative study of self-care strategies and social and professional support in early adolescence. J Early Adolesc. (2020) 40(5):605–32. 10.1177/0272431619858420

[5] Stapley E Eisenstadt M Demkowicz O Garland L Stock S Deighton J. *Shining a light on risk and protective factors: Young people’s experiences*. (2020). Available online at: https://www.ucl.ac.uk/evidence-based-practice-unit/sites/evidence-based-practice-unit/files/evidence_briefing_6_january_2020.pdf (Accessed February 21, 2023).

[6] Abate BB Sendekie AK Tadesse AW Engdaw T Mengesha A Zemariam AB Resilience after adversity: an umbrella review of adversity protective factors and resilience-promoting interventions. Front Psychiatry. (2024) 15:1391312. 10.3389/fpsyt.2024.1391312 39429523 PMC11487322

[7] Gartland D Riggs E Muyeen S Giallo R Afifi TO MacMillan H What factors are associated with resilient outcomes in children exposed to social adversity? A systematic review. BMJ Open. (2019) 9(4):e024870. 10.1136/bmjopen-2018-024870 30975671 PMC6500354

[8] Fritz J de Graaff AM Caisley H van Harmelen A-L Wilkinson PO. A systematic review of amenable resilience factors that moderate and/or mediate the relationship between childhood adversity and mental health in young people. Front Psychiatry. (2018) 9:230. 10.3389/fpsyt.2018.00230 29971021 PMC6018532

[9] Lynch SJ Sunderland M Newton NC Chapman C. A systematic review of transdiagnostic risk and protective factors for general and specific psychopathology in young people. Clin Psychol Rev. (2021) 87:102036. 10.1016/j.cpr.2021.102036 33992846

[10] Bluth K Roberson PNE Gaylord SA Faurot KR Grewen KM Arzon S Does self-compassion protect adolescents from stress? J Child Fam Stud. (2016) 25(4):1098–109. 10.1007/s10826-015-0307-3 26997856 PMC4793986

[11] Fergus S Zimmerman MA. Adolescent resilience: a framework for understanding healthy development in the face of risk. Annu Rev Public Health. (2005) 26(1):399–419. 10.1146/annurev.publhealth.26.021304.144357 15760295

[12] Hamby S Grych J Banyard V. Resilience portfolios and poly-strengths: identifying protective factors associated with thriving after adversity. Psychol Violence. (2018) 8(2):172–83. 10.1037/vio0000135

[13] Hamby S Taylor E Jones L Mitchell KJ Turner HA Newlin C. From poly-victimization to poly-strengths: understanding the web of violence can transform research on youth violence and illuminate the path to prevention and resilience. J Interpers Violence. (2018) 33(5):719–39. 10.1177/0886260517744847 29411696

[14] Sharma S Mustanski B Dick D Bolland J Kertes DA. Protective factors buffer life stress and behavioral health outcomes among high-risk youth. J Abnorm Child Psychol. (2019) 47(8):1289–301. 10.1007/s10802-019-00515-8 30796646 PMC6616218

[15] Benner AD. The transition to high school: current knowledge, future directions. Educ Psychol Rev. (2011) 23(3):299–328. 10.1007/s10648-011-9152-0 21966178 PMC3182155

[16] Patton GC Olsson CA Skirbekk V Saffery R Wlodek ME Azzopardi PS Adolescence and the next generation. Nature. (2018) 554(7693):458–66. 10.1038/nature25759 29469095

[17] Gervais C Jose PE. Relationships between family connectedness and stress-triggering problems among adolescents: potential mediating role of coping strategies. Res Child Adolesc Psychopathol. (2024) 52(2):237–51. 10.1007/s10802-023-01122-4 37725201

[18] Luthar SS Sawyer JA Brown PJ. Conceptual issues in studies of resilience: past, present, and future research. Ann N Y Acad Sci. (2006) 1094(1):105–15. 10.1196/annals.1376.009 17347344 PMC3480733

[19] Stapley E Stock S Deighton J Demkowicz O. A qualitative study of how adolescents’ use of coping strategies and support varies in line with their experiences of adversity. In: Weems CF, editor. Child & Youth Care Forum. New York: Springer (2023), Vol. 52, (1), p. 177–203. 10.1007/s10566-022-09682-0 PMC8886192 35250250

[20] Bishop L. A reflexive account of reusing qualitative data: beyond primary/secondary dualism. Sociol Res Online. (2007) 12(3):43–56. 10.5153/sro.1553

[21] Willig C. Perspectives on the epistemological bases for qualitative research. In: Cooper H, editor. APA Handbook of Research Methods in Psychology: Foundations, Planning, Measures, and Psychometrics. Washington, DC: American Psychological Association (2012), Vol. 1, p. 5–21. 10.1037/13619-002

[22] Molenaar PCM. Personalized models of psychological processes: idiographic research in the age of big data. J Child Psychol Psychiatry. (2017) 58(3):274–7. 10.1111/jcpp.12672

[23] Stapley E Eisenstadt M Demkowicz O Stock S O’Neill A Deighton J Early adolescents’ experiences of a school- and community-based prevention program: perceived ‘bridges’ and ‘walls’ to promoting mental health and wellbeing. Adv Ment Health. (2023) 22(1):82–103. 10.1080/18387357.2023.2210704

[24] O'Neill A Stapley E Stock S Merrick H Humphrey N. Adolescents’ understanding of what causes emotional distress: a qualitative exploration in a non-clinical sample using ideal-type analysis. Front Public Health. (2021) 9:673321. 10.3389/fpubh.2021.673321 34109149 PMC8181134

[25] Copeland-Linder N Lambert SF Ialongo NS. Community violence, protective factors, and adolescent mental health: a profile analysis. J Clin Child Adolesc Psychol. (2010) 39(2):176–86. 10.1080/15374410903532601 20390809 PMC3584688

[26] Solberg VSH Carlstrom AH Howard KAS Jones JE. Classifying at-risk high school youth: the influence of exposure to community violence and protective factors on academic and health outcomes. Career Dev Q. (2007) 55(4):313–27. 10.1002/j.2161-0045.2007.tb00086.x

[27] Ungar M Hadfield K Ikeda J. Adolescents' experiences of therapeutic relationships at high and low levels of risk and resilience. J Soc Work Pract. (2017) 32(3):277–92. 10.1080/02650533.2017.1384999

[28] Laursen BP Hoff E. Person-centered and variable-centered approaches to longitudinal data. Merrill Palmer Q. (2006) 52(3):377–89. 10.1353/mpq.2006.0029

[29] Stattin H Eriksson C. Person-oriented profiles can clarify Variable-oriented associations: the example of communication with parents and adolescents’ mental health problems. Youth. (2024) 4(1):42–55. 10.3390/youth4010004

[30] Kazdin AE Kraemer HC Kessler RC Kupfer DJ Offord DR. Contributions of risk-factor research to developmental psychopathology. Clin Psychol Rev. (1997) 17(4):375–406. 10.1016/S0272-7358(97)00012-3 9199858

[31] Evidence Practice Unit. *HeadStart national evaluation final report executive summary.* (2023). Available online at: https://www.ucl.ac.uk/evidence-based-practice-unit/sites/evidence_based_practice_unit/files/headstart_master_exec_summary.pdf (Accessed June 07, 2024).

[32] Kvale S Brinkmann S. Interviews: Learning the Craft of Qualitative Research Interviewing. 3rd ed. Thousand Oaks, CA: Sage (2015). p. 405.

[33] Weber M. Objectivity’ in social science and social policy. In: Shils EA Finch HA, editors. The Methodology of the Social Sciences. New York, NY: Free Press (1949). p. 49–112.

[34] Gerhardt U. The use of weberian ideal-type methodology in qualitative data interpretation: an outline for ideal-type analysis. Bull Sociol Methodol. (1994) 45(1):74–126. 10.1177/075910639404500105

[35] Stapley E O’Keeffe S Midgley N. Developing typologies in qualitative research: the use of ideal-type analysis. Int J Qual Methods. (2022) 21:1. 10.1177/16094069221100633

[36] Werbart A Grünbaum C Jonasson B Kempe H Kusz M Linde S Changes in the representations of mother and father among young adults in psychoanalytic psychotherapy. Psychoanal Psychol. (2011) 28(1):95–116. 10.1037/a0022344

[37] Stapley E O’Keeffe S Midgley N. Essentials of Ideal-Type Analysis: A Qualitative Approach to Constructing Typologies. Washington, DC: American Psychological Association (2021). 10.1037/0000235-000

[38] Kühnlein I. Psychotherapy as a process of transformation: analysis of posttherapeutic autobiographic narrations. Psychother Res. (1999) 9(3):274–87. 10.1080/10503309912331332761

[39] Clark DB Cornelius JR Kirisci L Tarter RE. Childhood risk categories for adolescent substance involvement: a general liability typology. Drug Alcohol Depend. (2005) 77(1):13–21. 10.1016/j.drugalcdep.2004.06.008 15607837

[40] Pinkerton J Dolan P. Family support, social capital, resilience and adolescent coping. Child Fam Soc Work. (2007) 12(3):219–28. 10.1111/j.1365-2206.2007.00497.x

[41] Yu X Kong X Cao Z Chen Z Zhang L Yu B. Social support and family functioning during adolescence: a two-wave cross-lagged study. Int J Environ Res Public Health. (2022) 19(10):6327. 10.3390/ijerph19106327 35627864 PMC9140348

[42] Bottrell D. Understanding ‘marginal’ perspectives: towards a social theory of resilience. Qual Soc Work. (2009) 8(3):321–39. 10.1177/1473325009337840

[43] González-Carrasco M Vaqué C Malo S Crous G Casas F Figuer C. A qualitative longitudinal study on the well-being of children and adolescents. Child Indic Res. (2019) 12(2):479–99. 10.1007/s12187-018-9534-7

[44] Cutrona C. Social Support in Couples: Marriage as a Resource in Times of Stress. Thousand Oaks, CA, London and New Delhi: SAGE (1996). 10.4135/9781483327563

[45] McGrath B Brennan MA Dolan P Barnett R. Adolescent well-being and supporting contexts: a comparison of adolescents in Ireland and Florida. J Community Appl Soc Psychol. (2009) 19(4):299–320. 10.1002/casp.998

[46] Yoshikawa H. Prevention as cumulative protection: effects of early family support and education on chronic delinquency and its risks. Psychol Bull. (1994) 115(1):28–54. 10.1037/0033-2909.115.1.28 8310099

[47] O’Keeffe S Martin P Target M Midgley N. I just stopped going’: a mixed methods investigation into types of therapy dropout in adolescents with depression. Front Psychol. (2019) 10:75. 10.3389/fpsyg.2019.00075 30804827 PMC6370696

[48] Haavik L Joa I Hatloy K Stain HJ Langeveld J. Help seeking for mental health problems in an adolescent population: the effect of gender. J Ment Health. (2019) 28(5):467–74. 10.1080/09638237.2017.1340630 28719230

[49] Suka M Yamauchi T Sugimori H. Help-seeking intentions for early signs of mental illness and their associated factors: comparison across four kinds of health problems. BMC Public Health. (2016) 16(1):301. 10.1186/s12889-016-2998-9 27056546 PMC4825081

[50] Hong J-C Hwang M-Y. Gender differences in help-seeking and supportive dialogue during on-line game. Procedia Soc Behav Sci. (2012) 64:342–51. 10.1016/j.sbspro.2012.11.041

[51] Seiffge-Krenke I Persike M. Gendered pathways to young adult symptomatology: the impact of managing relationship stress during adolescence. Int J Behav Dev. (2017) 41(1):52–63. 10.1177/0165025416646485

[52] Paananen R Ristikari T Merikukka M Gissler M. Social determinants of mental health: a Finnish nationwide follow-up study on mental disorders. J Epidemiol Community Health. (2013) 67(12):1025–31. 10.1136/jech-2013-202768 23908462

[53] Triana R Keliat BA Sulistiowati NMD. The relationship between self-esteem, family relationships and social support as the protective factors and adolescent mental health. Humanit Soc Sci Rev. (2019) 7(1):41–7. 10.18510/hssr.2019.715

[54] Cleveland MJ Feinberg ME Bontempo DE Greenberg MT. The role of risk and protective factors in substance use across adolescence. J Adolesc Health. (2008) 43(2):157–64. 10.1016/j.jadohealth.2008.01.015 18639789 PMC2518980

[55] Cross D Barnes A Papageorgiou A Hadwen K Hearn L Lester L. A social–ecological framework for understanding and reducing cyberbullying behaviours. Aggress Violent Behav. (2015) 23:109–17. 10.1016/j.avb.2015.05.016

[56] Newcomb ME Heinz AJ Birkett M Mustanski B. A longitudinal examination of risk and protective factors for cigarette smoking among lesbian, gay, bisexual, and transgender youth. J Adolesc Health. (2014) 54(5):558–64. 10.1016/j.jadohealth.2013.10.208 24388111 PMC3999176

[57] Zolkoski SM Bullock LM. Resilience in children and youth: a review. Child Youth Serv Rev. (2012) 34(12):2295–303. 10.1016/j.childyouth.2012.08.009

[58] McGorry PD Hartmann JA Spooner R Nelson B. Beyond the “at risk mental state” concept: transitioning to transdiagnostic psychiatry. World Psychiatry. (2018) 17(2):133–42. 10.1002/wps.20514 29856558 PMC5980504

[59] Hardt J Rutter M. Validity of adult retrospective reports of adverse childhood experiences: review of the evidence. J Child Psychol Psychiatry. (2004) 45(2):260–73. 10.1111/j.1469-7610.2004.00218.x 14982240

[60] Krumpal I. Determinants of social desirability bias in sensitive surveys: a literature review. Qual Quant. (2013) 47(4):2025–47. 10.1007/s11135-011-9640-9

[61] Leung L. Validity, reliability, and generalizability in qualitative research. J Family Med Prim Care. (2015) 4(3):324. 10.4103/2249-4863.161306 26288766 PMC4535087

[62] Smith B. Generalizability in qualitative research: misunderstandings, opportunities and recommendations for the sport and exercise sciences. Qual Res Sport Exerc Health. (2018) 10(1):137–49. 10.1080/2159676X.2017.1393221

